# Efficacy of Oral Tofacitinib in Alopecia Areata, Alopecia Totalis, and Alopecia Universalis

**DOI:** 10.7759/cureus.82536

**Published:** 2025-04-18

**Authors:** Syeda Shahmoona Tirmizi, Mahnoor Khalil Ahmed, Tayyaba Iqbal, Atif A Hashmi

**Affiliations:** 1 Dermatology, Hamdard Medical University, Karachi, PAK; 2 Dermatology, Karachi Metropolitan University, Karachi, PAK; 3 Dermatology, Dow University of Health Sciences, Karachi, PAK; 4 Pathology, Liaquat National Hospital and Medical College, Karachi, PAK

**Keywords:** alopecia areata, alopecia totalis, alopecia universalis, autoimmune hair disorder, tofacitinib

## Abstract

Objectives

Tofacitinib, an effective Janus kinase inhibitor (JAKi), has gained increasing interest in recent years for the management of refractory alopecia areata (AA). One of the most prevalent autoimmune diseases is AA, a kind of non-scarring alopecia. Therefore, the purpose of this study was to evaluate the efficacy of oral tofacitinib in treating AA, alopecia totalis (AT), and alopecia universalis (AU).

Methodology

This interventional study was conducted at Hamdard Medical University, Taj Medical Complex, using a non-probability consecutive sampling technique. The duration of the study was about six months. This study included 50 patients of both genders diagnosed with AA, AT, and AU, aged five years to 50 years, and who were fit for treatment with oral tofacitinib. Pediatric patients received 5 mg once daily, while adults received 5 mg twice daily. Treatment response was assessed at eight, 12, and 24 weeks using changes in the Severity of Alopecia Tool (SALT) score from baseline. A Chi-square test was used to compare SALT scores and percentage changes over the follow-up period.

Results

The study findings showed that the mean age of the patients was 25.6 ± 11.8 years. Of them, 23 (46.0%) were males, and 27 (54.0%) were females. The majority of the patients (39, 78.0%) had AA, with nine (18.0%) patients having AU, and two (4.0%) having AT. The mean (SD) pretreatment scalp hair loss was 62.48 ± 23.58 %, and the mean scalp involvement at 24-week follow-up was 10.5 ± 24.0%. The mean regrowth rate was 88.9 ± 24.5 %. Moreover, a statistically significant difference was observed between the changes in SALT score from baseline and the scores of SALT at eight, 12, and 24 weeks (p < 0.001).

Conclusion

This study concluded that administration of oral tofacitinib to AA patients had significantly improved hair growth. Additionally, it has been revealed to be a potentially efficacious therapy for the management of severe and refractory disease. However, our follow-up period was small, and some side effects, such as changes in lipid count and cardiovascular side effects, may take a longer time to develop; therefore, longer follow-up studies are needed to better evaluate the long-term safety of this drug in AA.

## Introduction

Alopecia areata (AA) is one of the most prevalent autoimmune diseases, with a 1.7% risk for lifetime [1,2]. It is a non-scarring alopecia that causes hair cycle stagnation in the anagen stage due to the lack of immunological reserve in hair follicles [3]. Alopecia totalis (AT) and alopecia universalis (AU) cause a complete lack of scalp and body hair, respectively, and an ophiasis pattern. AA causes loss of hair restricted to the temporal and occipital regions of the scalp. The autoimmune mechanism that causes hair loss is thought to cause persistent inflammation because it is accompanied by an organ-specific CD8+ T-cell-dependent response that primarily affects hair follicles [4].

Generally, 2% of people will experience AA at some point in their lives [5]. The average beginning of AA occurs between the ages of 25 and 36, and the condition's incidence seems to increase almost linearly with age [6]. The early-onset AA subtype usually manifests with more severe symptoms in children below the age of 10 [7]. It has been associated with an increased incidence of lupus erythematosus, vitiligo, and autoimmune thyroid disease, among other autoimmune illnesses [8].

The etiological factor of AA is an intricate connection between the immune system and genetic defects that leads to inflammation that specifically targets hair follicles. It is believed that the precursor for AA is the breakdown of the immunological reserve of hair follicles [9]. The primary components in the pathophysiology of AA have been found to be CD8 + NKG2D + T cells and interferon-γ (IFN-γ) [10]. Research on animal models of AA has demonstrated that CD8 + NKG2D + T cells release IFN-γ through processes involving Janus kinase (JAK)1/2, which in turn stimulates the generation of interleukin-15 (IL-15) in follicular epithelial cells. After binding to the surface of CD8 + NKG2D + T cells, IL-15 intensifies the response of inflammatory cells and disrupts the hair growth cycle by stimulating the production of IFN-γ through JAK1/3 systems [11].

JAK inhibitors, often known as JAKi, have been researched as a potential treatment for AA due to the significant role that JAK pathways play in the pathophysiology of the disease. This treatment strategy may inhibit many signaling pathways, which would result in a reduction in CD8 + NKG2D + T cells and a notable improvement in AA. Several JAKi, such as tofacitinib (JAK1/3), ruxolitinib (JAK1/2), and baricitinib (JAK1/2), have been described to date for the treatment of AA [12]. Tofacitinib monotherapy has shown remarkable success in a number of case reports and studies, which may increase the range of therapeutic choices available for moderate-to-severe AA [13].

Tofacitinib's efficacy in treating AA, AT, and AU has been the subject of recent research. With notable improvements in disease progression and hair regeneration, such investigations have demonstrated encouraging outcomes [14]. For 24 weeks, individuals with moderate to severe AA received either tofacitinib or a placebo in a randomized, double-blind, placebo-controlled study. According to the study, just 9.5% of patients in the placebo group experienced hair regrowth of at least 50%, while 58% of patients in the tofacitinib group did. Both disease activity and quality of life significantly improved in the tofacitinib group [1].

A different study evaluated tofacitinib's efficacy in treating people with AT or AU. After receiving tofacitinib treatment for six months, the study found that 75% of the patients experienced appreciable hair growth. According to the study, both disease activity and quality of life improved [15].

Studies demonstrating the efficacy of tofacitinib and ruxolitinib in the therapeutic management of AU are scarce in the literature [1,16]. Although serious side effects of tofacitinib were noted, including cardiovascular side effects, increased lipid levels, and increased risk of infections, the drug is approved for protracted use in many immune-mediated diseases, including ulcerative colitis [17]. According to a small number of studies that recommend maintenance therapy after recovery, one of the medication's drawbacks is that the medicine can cause the condition to develop or reappear after quitting it [1,18]. Moreover, these side effects were mainly observed when this drug was used in the treatment of refractory autoimmune diseases. Nevertheless, strict selection criteria are needed before starting the drug. 

Tofacitinib appears to be a potential therapy choice for AT, AU, and AA based on the evidence that is currently available. The studies have demonstrated notable improvements in disease activity and hair regeneration. Thus, the purpose of this study was to evaluate the efficacy of oral tofacitinib in individuals suffering from AA, AT, and AU. This article was previously presented as a meeting abstract at the Pakistan Association of Dermatologists Conference (PADCON) on November 18, 2023.

## Materials and methods

This interventional study was conducted at Hamdard Medical University, Taj Medical Complex, using a non-probability consecutive sampling technique. This study was approved by the Institutional Review Board of the concerned hospital. The study duration was about six months after obtaining the approval of the synopsis. The study included 50 patients of both genders diagnosed with AA, AT, and AU, aged five years to 50 years, and who were fit for treatment with oral tofacitinib. After the evaluation of patients, a 5 mg once-daily dose in the pediatric group and a twice-daily dose of oral tofacitinib in adults were started. In addition, the study excluded those who refused to give their agreement to participate, had a history of oral tofacitinib hypersensitivity, or were using topical tofacitinib.

The study was approved by the ethical review committee of Hamdard College of Medicine and Dentistry (HCM & D/687/2023). Following ethical approval, patients underwent a thorough history and general and systemic examinations to evaluate their condition. All cases included in the study had moderate to severe disease, refractory to other forms of treatment, including steroids. Before starting the drug, tuberculosis screening was performed to exclude active tuberculous disease, including QuantiFERON Gold (IGRA), Montoux test, and erythrocyte sedimentation rate (ESR). In addition, baseline, complete blood count (CBC), liver function tests (LFTs), and serum creatinine were performed. Hepatitis B and C and HIV serologies were done to exclude these infections. Immunocompromised patients, patients with a history of cancer, and ongoing infection were excluded from the study. 

Following a patient evaluation, either a 5 mg twice-daily or a 10 mg once-daily oral tofacitinib dosage was initiated. We titrated the dose based on response and tolerability. At eight, 12, and 24 weeks following the initiation of treatment, the effectiveness of the treatment was evaluated. Utilizing the Severity of Alopecia Tool (SALT) to validate the initial response time, a higher score indicating more severe disease, was defined as the time to the first recorded sign of any hair growth [19]. By calculating the percentage of hair loss in the four sections of the scalp (vertex: 40%), right profile: 18%, left profile: 18%, and posterior: 24%), and summing the results, one can calculate the SALT score. SALT scoring was carried out both before and over the course of follow-ups. The formula for calculating the percentage change in SALT score from the baseline was initial SALT score-best SALT score on treatment/initial SALT score x 100%.

While MCID is useful in some contexts, SALT remains the gold standard for AA trials due to its objective, validated nature and broad clinical acceptance. Since SALT measures disease severity continuously, it avoids subjective patient-reported biases that could affect minimum clinically important difference (MCID) calculations. We used a cut-off SALT score of 50, based on previous studies [20].

Data analysis

The data was analyzed using IBM SPSS Statistics for Windows, Version 23 (Released 2015; IBM Corp., Armonk, New York, United States). Quantitative variables were presented as mean and standard deviation, while qualitative variables were documented as frequencies and percentages. The paired t-test was used to compare the mean scores of baseline hair loss and follow-up after therapy at 24 weeks. Fisher's exact test was applied to determine the association among the variants of AA and the change in SALT score. A p-value of <0.05 is reflected as statistically significant.

## Results

A total of 50 patients with AA were included in the study, with a mean age of 25.64 ± 11.77 years. Of them, 23 (46.0%) were males, and 27 (54.0%) were females. The mean duration of disease was 44.94 ± 64.77 months, and the mean duration of therapy was 7.78 ± 3.38 weeks. Most of the patients belonged to the middle class (45, 90.0%). The most common variant of AA was AA (39, 78.0%), AU (9, 18.0%), and AT (2, 4.0%). The mean baseline score of scalp involvement was 62.48 ± 23.58 %, and the mean final follow-up score of scalp involvement was 10.46 ± 24.04 %, while the change in SALT score was 88.64 ± 24.64%. Moreover, the mean regrowth of hairs was 88.90 ± 24.52 %. Additionally, there were no adverse events reported by any patients treated with oral tofacitinib, as shown in Table 1.

**Table 1:** Demographic details of patients with alopecia areata treated with oral tofacitinib (n = 50) SALT: Severity of Alopecia Tool

Variable		Mean ± SD, n(%)
Age (years)		25.64 ± 11.77
Duration of disease (months)		44.94 ± 64.77
Duration of therapy (weeks)		7.78 ± 3.38
Mean pretreatment scalp hair loss (%)		62.48 ± 23.58
Scalp Involvement score at follow-up (%)		10.46 ± 24.04
Change in SALT score (%)		88.64 ± 24.64
Regrowth (%)		88.90 ± 24.52
Gender	Male	23 (46.0%)
	Female	27 (54.0%)
Socioeconomic status	High	4 (8.0%)
	Middle	45 (90.0%)
	Low	1 (2.0%)
Diagnosis	Alopecia areata	39 (78.0%)
	Alopecia totalis	2 (4.0%)
	Alopecia universalis	9 (18.0%)
Adverse events	No significant side effects	50 (100.0%)

**Figure 1:**
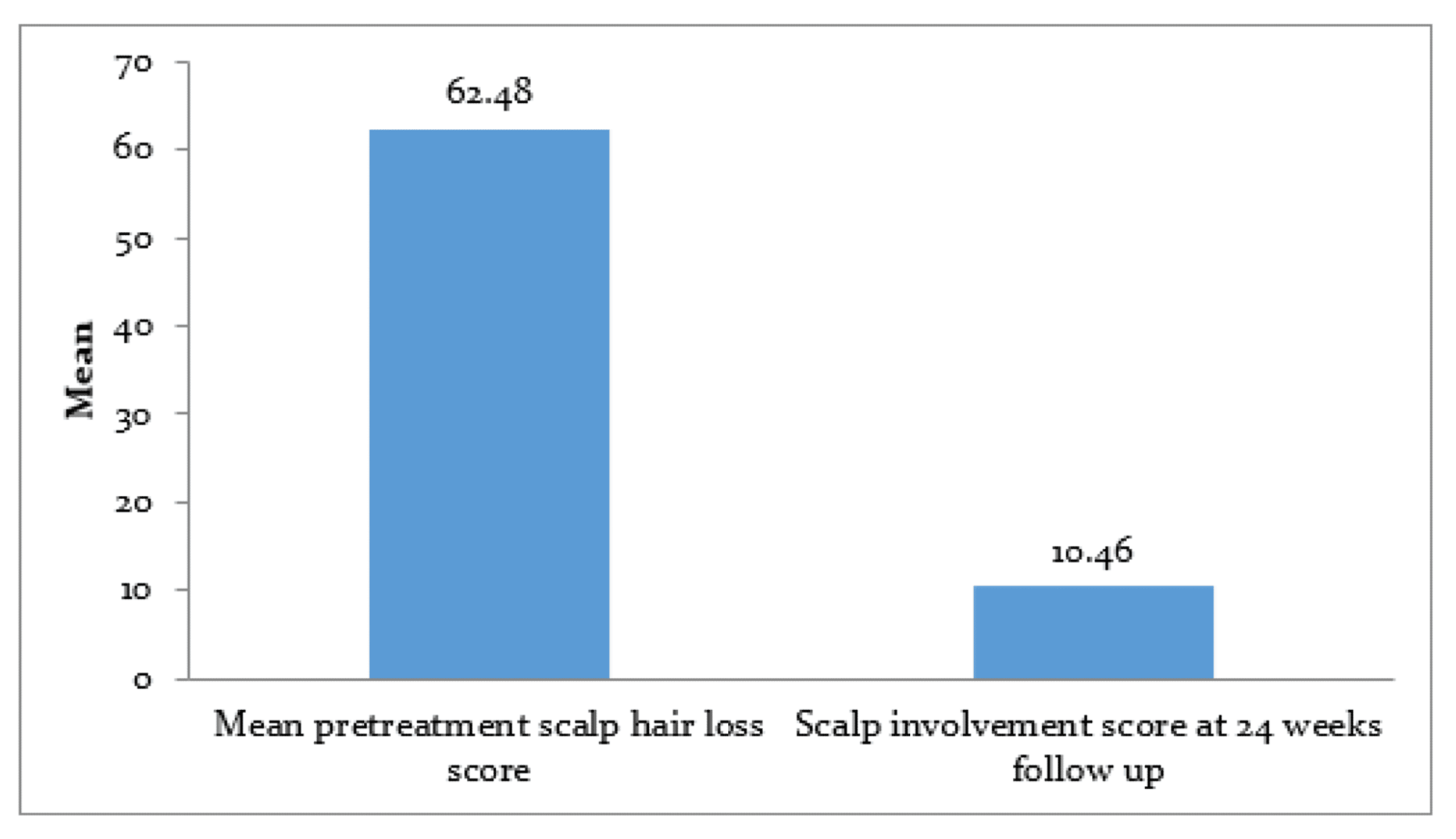
The comparison of baseline score (62.3 + 23.6) and follow-up score at 24 weeks

The comparison of the baseline score and follow-up score at 24 weeks revealed a significant difference with respect to scalp involvement. It was observed that there was a significant association between mean pretreatment scalp hair loss (62.48 ± 23.58), and the 24-week follow-up score (10.46 ± 24.04) (p < 0.001), as shown in Figure 1.

Regarding the improvement in AA patients, it was observed that 39 (100.0%) patients with AA had >50% highest percent change in SALT score, while patients with AT (2, 100.0%) showed >50% change in SALT score, and patients with AU (5, 55.6%) showed >50% change in SALT score, and only 4 (44.4%) showed <50% change in SALT score, with a significant association found between them (p = 0.009), as shown in Table 2.

**Table 2:** Improvement in AA patients according to the change in SALT score AA: alopecia areata; SALT: Severity of Alopecia Tool *p-value significant as < 0.05. Fisher's exact test was applied

Variable								
		<50 (%)	>50 (%)	Total n (%)	Test statistics (Fisher’s exact test)	df	p-value	
Type of alopecia areata	Alopecia areata	0 (0.0%)	39 (100.0%)	39 (100.0%)	13.9	2	0.001*	
	Alopecia totalis	0 (0.0%)	2 (100.0%)	2 (100.0%)				
	Alopecia universalis	4 (44.4%)	5 (55.6%)	9 (100.0%)				
Total		4 (8.0%)	46 (92.0%)	50 (100.0%)				

**Figure 2:**
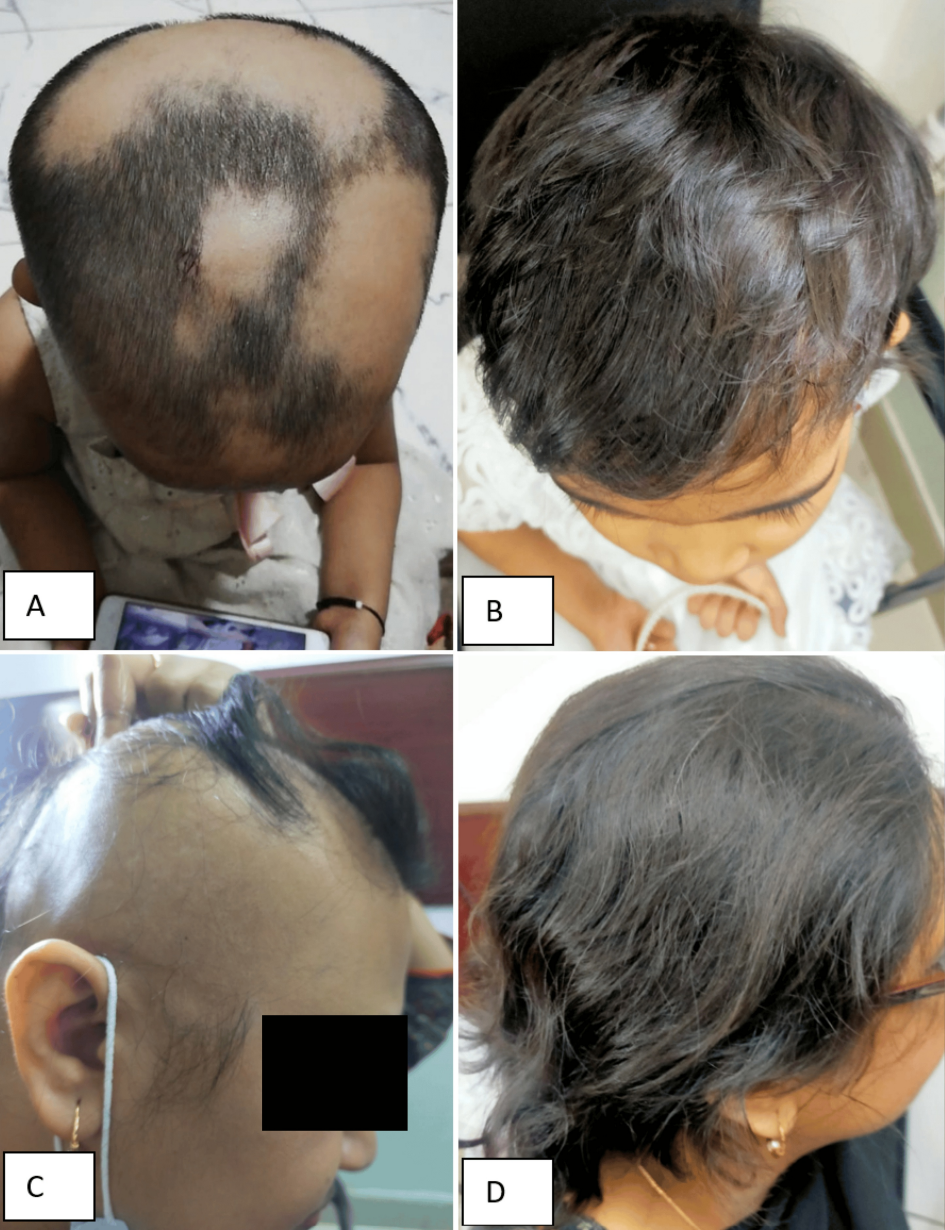
Pretreatment and posttreatment images of patients with alopecia areata. (A & C) pretreatment, (B & D) 24-weeks post-treatment

 A few cases of AA were shown in Figure 2 (before and after the treatment). 

## Discussion

AA is a prevalent cause of hair loss in both children and adults. For adult patients with AA, recent research has shown that tofacitinib, a JAKi, is both safe and effective [21]. Therefore, this study demonstrated the efficacy of oral tofacitinib in children and adult patients with AA, AT, and AU.

In Pakistan, an interventional study evaluated 60 individuals with AA. For a duration of 24 weeks, patients received tofacitinib 5 mg twice daily. The patients' ages were 33.70 ± 8.69 years. Patients with AA had a change in SALT score of above 50% in 82.9% of cases, patients with AT in 75% of cases, and patients with AU in 66.7% of cases. The study's findings indicated that tofacitinib is an effective therapy for AA [22]. Their findings demonstrated that giving AA patients 5 mg of tofacitinib twice daily for six months can lead to improvement [22]. These results were consistent with a number of investigations carried out in other nations [3,23,24]. This study was in line with the previously published research, revealing that the mean age of the patients was 25.64 ± 11.77 years, and all the patients with AA and AT showed >50% change in SALT score, while patients with AU (5, 55.6%) showed >50% change in SALT score, and only four (44.4%) showed <50% change in SALT score, showing the promising outcomes of the tofacitinib therapy in patients with AA. 

Similarly, another study found that 58.3% of individuals had AA, 26.7% had AT, and 15% had AU. The most prevalent variant was AA [22]; these results were consistent with other studies that found that most of their patients had the AA variation [25]. These findings were similar to this study and revealed that AA was the most common type of variant observed and was diagnosed in 39 (78.0%) patients, followed by AU (9, 18.0%), and AT (2, 4.0%).

A clinical trial in Bangladesh evaluated the safety and effectiveness of tofacitinib for AA treatment in 45 patients with >50% scalp involvement. The average patient age was 33 years, with 40% under 20. The study included 24.4% with AT, 17.8% with AU, and 57.8% with patchy AA. The disease duration averaged 1.2 years. Among those with patchy alopecia, 84.6% achieved full hair regrowth, while 15.4% had partial regrowth. No adverse effects were reported by 75.6% of patients, while 24.4% experienced side effects, including nausea (18.2%), headaches (72.7%), and severe respiratory infections (9.1%). Tofacitinib and JAKi were concluded to be safe, reliable, and effective treatments for AA [26]. These findings were partially consistent with this study and revealed that the mean age of the patients was 25.64 ± 11.77 years, and the mean duration of disease was 44.94 ± 64.77 months. The most common variant of AA was AA (39, 78.0%), AU (9, 18.0%), and AT (2, 4.0%). Moreover, the mean regrowth of hairs was 88.90 ± 24.52%. Regarding the improvement in AA patients, it was observed that 39 (100.0%) patients with AA had >50% highest percent change in SALT score. Additionally, there were no adverse events reported by any patients treated with oral tofacitinib.

Likewise, another retrospective study demonstrates the efficacy of tofacitinib in pediatric AA. In total, 82% of patients (9/11) reported hair growth, and 64% (7/11) reported a score improvement of more than 50% on the SALT. Based on changes in their SALT scores, 87% (27/31) of these patients demonstrated substantial responses [21], which was greater than the change noted in adult patients [23,27] but comparable to the change described by others [28]. The course of therapy lasted from six to 18 months. The reported adverse effects during follow-up were moderate and manageable. Upper respiratory infection (URI) (14.7%), increased liver enzymes alanine aminotransferase/aspartate aminotransferase (14.7%), and eosinophilia (14.7%) were the most commonly reported adverse events [21]. These findings were partially in agreement with this study, showing that most patients experienced an improvement in hair growth following tofacitinib medication administration. The average percentage of hair growth was 88.90 ± 24.52%. Furthermore, none of the individuals receiving oral tofacitinib reported any adverse effects.

A retrospective study of 20 individuals with severe AA treated with tofacitinib included 90% female patients. The mean baseline SALT score was 88%, with 70% having AU and 20% AT. The average disease duration was 2.4 years. Twelve patients (60%) received treatment for at least 12 months, with treatment durations ranging from 0.5 to 28 months (average 13 months). Hair regrowth began after an average of 3.85 months, with 70% showing regrowth within three months. SALT score improved by more than 50% in 55% of patients, while 25% achieved complete regrowth (>90% SALT improvement). Among those treated for over 12 months, 91.7% experienced regrowth [23]. This study was in agreement with the abovementioned studies and reported that more than half of the patients diagnosed with AA were female (27, 54.0%). The mean duration of disease was 44.94 ± 64.77 months, and the mean duration of therapy was 7.78 ± 3.38 weeks, with a mean baseline hair loss of 62.48 ± 23.58%. About 39 (100.0%) patients with AA had >50% highest percent change in SALT score, while patients with AT (2, 100.0%) showed >50% change in SALT score.

A retrospective study conducted in North India analyzed 17 patients with AA who were prescribed tofacitinib 5 mg twice daily for six months. The average age of the participants was 27.88 ± 16.30 years, with a male-to-female ratio of 2.4:1. The disease had an average duration of 32.29 ± 22.14 months. Following six months of tofacitinib treatment, there was a significant reduction in the SALT score (p = 0.0001), indicating the drug’s efficacy in treating AA [29]. These findings align with the results of this study, where the mean patient age was 25.64 ± 11.77 years, and the average disease duration was 44.94 ± 64.77 months. Notably, all patients with AA and AT showed a significant (>50%) improvement in their SALT scores. Additionally, among patients with AU, five out of nine (55.6%) exhibited a >50% reduction in the SALT score (p = 0.009).

This study's limitations included the lack of a control group, which made it impossible to compare tofacitinib to a placebo, and the limited sample size, which made it impossible to perform subgroup analysis. Therefore, more extensive randomized controlled studies are required to determine the best course of treatment for preserving response and minimizing hazards. This study had a short follow-up duration. Some side effects take longer than 24 weeks to manifest (e.g., thromboembolic events or lipid changes). Long-term side effects, such as increased cardiovascular risk, might not be evident within a short study timeframe.

## Conclusions

This study concluded that administration of oral tofacitinib to AA patients had significantly improved hair growth. Additionally, it has been revealed to be a potentially efficacious therapy for the management of severe, refractory disease. However, our follow-up period was small, and some side effects, such as changes in lipid count and cardiovascular side effects, may take a longer time to develop. Consequently, larger, more comprehensive randomized clinical trials are necessary to further test the long-term safety and therapeutic efficacy of tofacitinib for the treatment of this disease.
